# Clinical Notes on Sepia

**Published:** 1885-04

**Authors:** 


### CLINICAL NOTES ON SEPIA.

She often feels as though she could feel every muscle and fibre of her right side from shoulder to the feet. It is an indescribable sensation.

During first sleep she often imagines she has swallowed something which wakes her up in a fright, with a sensation as though it had lodged in her throat. This sensation remains after waking.

Cramp in calves and feet, mostly in daytime, and her ankles are weak and turn easily when walking, so that she stumbles. Urine scanty, and deposits milky substance.

Sensation as of a strap, as wide as her hand, tight around her waist, in evening after supper.

Beating at pit of stomach; sour stomach; fiery zigzag before eyes; metallic taste in mouth; cracking in knee-joints, and painful cracking in occiput.

Frequent sensation, as if a knife were thrust into the apex of the left lung, with pain streaking off through the shoulder.

Sensation in both hypochondria, as though the ribs were broken, and sharp points were sticking in the flesh.—*H. N. M. in Jour. of M. M.*

